# Oral health-related quality of life in the LGBTIQ+ population: a cross-sectional study

**DOI:** 10.1590/1807-3107bor-2024.vol38.0041

**Published:** 2024-05-13

**Authors:** Luiz Eduardo de ALMEIDA, Pablo Fellipe de Souza ALMEIDA, Valéria de OLIVEIRA, Fábio Luiz MIALHE

**Affiliations:** (a) Universidade Federal de Juiz de Fora – UFJF, School of Dentistry, Department of Restorative Dentistry, Juiz de Fora, MG, Brazil.; (b) Instituto Federal de Educação, Ciência e Tecnologia do Sudeste de Minas Gerais, School of Computer Science, Rio Pomba, MG, Brazil.; (c) Universidade Federal de Juiz de Fora – UFJF, Institute of Life Sciences, Department of Dentistry, Governador Valadares, MG, Brazil.; (d) Universidade Estadual de Campinas – Unicamp, School of Dentistry, Department of Health Sciences and Children’s Dentistry, Piracicaba, SP, Brazil.

**Keywords:** Quality of life, Oral Health, Sexual and Gender Minorities, Epidemiology, Cross-Sectional Studies

## Abstract

The aim of this cross-sectional study was to investigate the associations between oral health-related quality of life (OHRQoL) and socioeconomic and demographic variables, suicidal ideation, self-perception of oral health, and experiences of dental care in the Brazilian adult LGBTIQ+ population. A sample of 464 participants completed self-administered online questionnaires and provided information for OHRQoL assessment, using the OHIP-14 instrument at three hierarchical levels of explanatory variables: LGBTIQ+ identities; socioeconomic and demographic data and existential suffering; and self-perception of oral health and experience of dental care. The collected data were fitted to hierarchical multiple logistic regression models, in which the associations between each independent variable with the OHIP-14 prevalence outcome were analyzed. The OHIP-14-prevalence index showed that 33.2% of the participants answered ‘frequently’ or ‘always’, and the highest frequencies were obtained for the psychological discomfort (27.8%), psychological disability (18.3%), and physical pain (17.5%) domains. According to the adjusted final model, LGBTIQ+ individuals who were more likely to have their OHRQoL affected were those who were indifferent (OR=3.21; 95% CI: 1.26-8.20), dissatisfied (OR=10.45; 95% CI: 3.86-28.26), or very dissatisfied (OR=53.93; 95% CI: 12.12-239.93) with their oral health status, and also those who had or have difficulty accessing dental treatment (OR=2.06; 95% CI: 1.24-3.41) (p<0.05). It may be concluded that the OHRQoL of the investigated Brazilian LGBTIQ+ population showed associations with individual aspects and with access to dental services.

## Introduction

According to the World Health Organization (WHO), “LGBTIQ+ health refers to the physical, mental, and emotional well-being of people who identify as lesbian, gay, bisexual, transgender, intersex, or queer (LGBTIQ+). The plus sign represents the vast diversity of people in terms of sexual orientation, gender identity, expression, and sex characteristics (SOGIESC).”^1^Worldwide estimates show a mismatch between the growing scientific understanding of LGBTIQ+ health needs and the evolution of health care for this population, given that a large part of these people still face pathologizing stigmas, discrimination, prejudice, and even violence that often persist within the health services they seek.^1,2^

WHO and the Pan American Health Organization (PAHO) highlight the importance of implementing public health policies for the vulnerable LGBTIQ+ population, giving special importance to the continuous improvement of care strategies and of general health indicators of these individuals.^1-3^

Studies have shown that LGBTIQ+ people contend with a disproportionate burden of adverse physical health outcomes and often experience discrimination, leading to decreased healthcare service utilization, poor self-perception of oral health, and worse oral health conditions compared to heterosexual individuals.^4-6^Together, these factors can lead to worse oral health-related quality of life (OHRQoL).^6^

The concept of OHRQoL is a multidimensional construct that encompasses functional, social, and emotional aspects and is related to the subjective assessment of the impact of oral health status on daily activities and on the well-being of individuals.^7,8^ Several instruments were developed to measure OHRQoL, among which, the oral health impact profile (OHIP) stands out for the fact that it evaluates several biopsychosocial domains.^7,8^

Until then, few studies had analyzed the OHRQoL of the LGBTIQ+ population worldwide,^9-10^ including Brazil.^11-12^ The only two studies published in Brazil to date were carried out with five LGBTIQ+ re-educated students from a prison in João Pessoa, state of Paraíba, and a controlled cross-sectional study conducted with 90 cross-dressing, trans, and transgender people in Uberlândia, state of Minas Gerais, comparing the OHRQoL of 45 cisgender and 45 transgender people.^11-12^Therefore, there is a need for studies with larger and more representative samples, as well as studies that investigate associations between socio-contextual variables and OHRQoL in this population, in order to fill these gaps in scientific production in Brazil.

Brazil is reported, even in the absence of accuracy associated with official data, as the most “LGBTIQ+phobic” country in the world, where it is estimated that an LGBTIQ+ is assaulted and killed, in that order, everyone and 27 hours.^13,14^ This context can lead to existential suffering and suicidal ideation in this population, affecting their quality of life.^15,16^ However, the implications of this aspect on the OHRQoL of this population are not known.

Therefore, the aim of this study was to investigate the associations between OHRQoL and socioeconomic and demographic variables, existential suffering, self-perception of oral health, and dental care experience in a sample of the adult LGBTIQ+ Brazilian population.

## Methodology

This is a cross-sectional observational study, whose development was guided by the recommendations of the STROBE (Strengthening the Reporting of Observational Studies in Epidemiology) initiative^17^and approved by the Research Ethics Committee of the Piracicaba Dental School (CAAE: 43945421.0.0000.5418).

The study was developed in Brazil and involved the application of self-administered questionnaires on an online environment (Google Forms^®^).

Study participants (adult LGBTIQ+ population with internet access) were recruited through five social networks (1. Instagram®: @saude.bucal.lgbtqiamais; 2. Facebook®: @saude. bucal.lgbtqia; 3. TikTok®: @saude.bucal.lgbtqia+; 4. Twitter®: @saude.bucal. lgbtqia+; and 5. WhatsApp ®: participation in interaction groups with LGBTQIA+ themes). Content about oral health and quality of life of the LGBTIQ+ population was periodically published on the aforementioned social networks in order to stimulate curiosity about the subject and to encourage the participation of the adult LGBTIQ+ Brazilian population in the study. In all posts on social media, there was an invitation letter that carried a link and QR code for interested parties to have access to the data collection instrument. In the aforementioned invitation letter, an access link (https://forms.gle/qZkMjeignjhjNw6j9) and QR code were provided for the participant to join the study. It is worth noting that the data collection process (from April 2021 to October 2022) relied on the participants’ reading and virtual knowledge of the informed consent form. Thus, before completing the data collection instruments, participants were informed about the estimated time (10 minutes) to answer the questionnaires and about the confidentiality and archiving of the data provided (all information collected was digitally archived in a virtual space, Google Drive®, highlighting the authors’ commitment to maintaining the anonymity of all participants, deleting the data collected 10 years after the date of publication of this study), in addition to providing the main researcher’s data (name, e-mail address, and telephone) to resolve any doubts. The data collection instruments were applied in “Google® Forms,” which allows the participant to return or proceed with the questionnaire-filling process, and conclude it by submitting the duly answered questionnaires. The total number of questions was 25 and the response time was approximately 10 minutes. The usability and technical functionality of the electronic questionnaire had been tested before fielding the questionnaire with three people from the LGTBIQA+ population, asking whether they had encountered any difficulties using and completing the questionnaire. No difficulties were reported by the participants regarding the aforementioned aspects. The eligibility criteria were minimum age of 18 years, self-identification as LGBTIQ+, and access to the internet.

The main outcome of the study was the OHRQoL^7,8^ of the LGBTIQ+ population, measured by the scores of the OHIP-14 questionnaire, which consists of 14 questions distributed across seven biopsychosocial domains.^7,8,14,18^This instrument offers five response options for each question, using a Likert scale (0 = never; 1 = rarely; 2 = sometimes; 3 = fairly often; 4 = very often).^7,8,14,18,19^ An established summary score or indicator was used, that is, prevalence of impacts, which represent the percentage of people responding ‘fairly often’ or ‘very often’ to one or more questions of the OHIP. Higher values denote poorer OHRQoL.^18,19^

The independent variables were organized considering their explanatory possibilities for the outcome of the study^10,11,16,18,19^ based on the minority stress model develop by Meyer.^20-22^ According to that model, socioeconomic factors and barriers to access services are considered examples of general stressors that affect the mental health of LGBTIQ+ people. Distal minority stressors include sexuality, color/race/ethnicity, and gender identity, and they can be associated with objective experiences of discrimination, oppression, and aggression (distal stress processes). Lastly, proximal stress processes are related to subjective processes or internal experiences and include self-critical beliefs, expectations of rejection, internalized homophobia, among others.^20^

In the present study, the variables were adapted to the model as follows:

**Level 1:** general stressors such as completed level of education (up to elementary, high school and/or technical, higher/college, and graduate), monthly family income in Brazilian minimum wages (BMW), which was later converted to U.S. dollars for the statistical analysis (1BMW ≈US$ 261), and accessibility to dental treatment (Have you had or do you have difficulty accessing dental treatment, that is, going to the dentist? / yes; no; I have never looked for and/or been to the dentist).^23^

**Level 2:** distal stress processes – included gender identity (how do you identify yourself? cisgender or transgender), sexual orientation (regarding your sexual orientation, how do you identify yourself? homosexual; heterosexual; bisexual; pansexual; asexual; other sexual identity), age (in years), color/race/ethnicity (white, brown, black, yellow/oriental/Japanese, indigenous, other), and suicidal ideation (have you ever thought about, planned, or tried to commit suicide / take your own life? (No; Yes, I just thought about it; Yes, I thought and planned; Yes, I thought, planned, and tried).^24-26^

**Level 3:** proximal stress processes – included the assessment of satisfaction with oral health status (regarding your teeth/mouth/oral health, what is your degree of satisfaction? very satisfied; satisfied; neither satisfied nor dissatisfied; dissatisfied; very dissatisfied) and self-perception of professional preparation to care for LGBTIQ+ patients (Do you believe that dentists are prepared to care for LGBTIQ+ patients? / yes; no)^4,27^(Figure).

**Figure:**
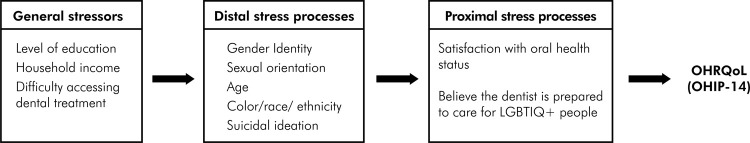
Conceptual model showing interconnected experiences that contribute to OHQOL of the LGBTIQ+ population (adapted from Meyer, 2003).^20^

For all the questions mentioned above, there was the answer option “I prefer not to answer or I do not know the answer.”

The sample size of the study involved the calculation of effect size, using the EpiInfo^TM^ software (version 7.2)^28^, and was based on parameters found in the collected data (rate of unexposed participants: 18%; minimum detectable odds ratio of 2.0 - information extracted from the outcome and the independent variable, in that order, “impact of OHRQoL” and “had or have difficulty accessing dental treatment”). Following this analytical approach, with a significance level of 5% (α = 0.05) and test power of 80% (β = 0.2), the minimum sample size was 398 participants.

Statistical analysis began with the descriptive evaluation of the variables. The data were then adjusted by logistic regression models to analyze the associations of each independent variable with the outcome (impact of OHRQoL).^29^ Variables with p < 0.20 were studied in hierarchical multiple logistic regression models.^29^ The variables were inserted in the model according to hierarchical levels, that is, the group of variables that make up the first level was the first to be inserted in the multiple model, followed by the group of variables at the second and third levels. The statistically significant variables of a hierarchical level were kept in the model and were analyzed together with the subsequent level, maintaining only the variables with p ≤ 0.05 in each model. The quality of adjustments was assessed using the Akaike information criterion (AIC).^29^ Unadjusted and adjusted odds ratios were estimated using model coefficients, with the respective 95% confidence intervals. In addition, we evaluated associations of cisgender and transgender people who answered ‘frequently’ or ‘always’ to one or more items of the OHIP-14, together with some outcomes, in order to understand in more detail how gender identity can affect them. For this purpose, the chi-square and Fisher’s exact tests were used. All these analyses were performed using the R^®^ statistical software.^30^

## Results

A total of 496 people participated in the study, of whom 32 (6.5%) were excluded 11 for being under 18 years of age and 21 for not fully completing the data collection instruments), thus totaling 464 LGBTIQ+ participants.

Most of the sample consisted of male (64.2%), cisgender (70.7%), and homosexual (55.4%) individuals (Table 1). The mean age of participants was approximately 30 years (standard deviation, SD = 10), 53.2% were white, 42.7 had finished high school, had a monthly family income of less than US$ 783 (48.3%), and only 38.6% never thought about, planned, or tried suicide. Less than half of the participants (46.1%) reported being satisfied or very satisfied with their oral health status (Table 1).

**Table 1:** Descriptive analysis of collected data (n = 464).

Category	Frequency ( )
Highest completed level of education	
No schooling	7 (1.5)
Elementary	23 (5.0)
High school and/or technical	198 (42.7)
Higher/college	90 (19.4)
Graduate	129 (27.8)
Did not answer / know	17 (3.6)
Household income US$	
Less than US$ 783	224 (48.3)
US$ 784 – US$ 1,305	81 (17.5)
US$ 1306 – US$ 1,827	55 (11.9)
US$ 1828 – US$ 2,610	50 (10.8)
More than US$ 2,610	51 (11.0)
Did not answer / know	3 (0.5)
Difficulty accessing dental treatment	
Yes	197 (42.5)
No	252 (54.3)
I never looked for and/or been to the dentist	15 (3.2)
Gender identity	
Cisgender	328 (70.7)
Transgender	136 (29.3)
Sexual orientation	
Heterosexual	42 (9.1)
Homosexual	257 (55.4)
Bisexual	96 (20.7)
Asexual	16 (3.4)
Pansexual	33 (7.1)
Other sexual identity	14 (3.0)
Did not answer	6 (1.3)
Age	
In full years	29.7 (18 - 70)
Color/race/ethnicity	
White	247 (53.2)
Brown	131 (28.2)
Black	68 (14.7)
Yellow/Oriental/Japanese	6 (1.3)
Indigenous	5 (1.1)
Other	7 (1.5)
Suicidal ideation	
No	179 (38.6)
Yes, I just thought about suicide	146 (31.4)
Yes, I thought about and planned suicide	50 (10.8)
Yes I thought about, planned, and tried suicide	72 (15.5)
Did not answer / know	17 (3.7)
Satisfaction with oral health status	
Very satisfied	52 (11.2)
Satisfied	162 (34.9)
Neither satisfied nor dissatisfied	136 (29.3)
Dissatisfied	82 (17.7)
Very dissatisfied	32 (6.9)
Believe the dentist is prepared to care for LGBTIQ+ persons	
Yes	106 (22.8)
No	189 (40.7)
Did not answer / know	169 (36.5)

Regarding dental experience, 42.5% reported that they had difficulties accessing dental treatment and only 22.8% of the sample believed that dentists were prepared to care for LGBTIQ+ patients (Table 1).

As for the OHRQoL, 33.2% of the participants answered ‘frequently’ or ‘always’ to one or more OHIP-14 items. Regarding the impact for each domain, it is important to highlight that “psychological discomfort (27.8%),” “psychological disability (18.3%),” and “physical pain (17.5%)” presented the highest frequencies (Table 2).

**Table 2:** OHRQoL of the LGBTIQ+ population according to OHIP-14 prevalence measure of impacts (responses rated as ‘fairly often’ or ‘very often’) and its domains (n = 464).

OHIP-14	Frequency	

	No impact n( )	With impact n( )
Prevalence of impacts	310 (66.8)	154 (33.2)
Domains		
Functional limitation	432 (93.1)	32 (6.9)
Physical pain	383 (82.5)	81 (17.5)
Psychological discomfort	335 (72.2)	129 (27.8)
Physical disability	432 (93.1)	32 (6.9)
Psychological disability	379 (81.7)	85 (18.3)
Social disability	432 (93.1)	32 (6.9)
Handicap	433 (93.3)	31 (6.7)

Table 3 presents the exploratory analysis of the collected data, from which statistically significant associations were verified between the OHIP-14 prevalence indicator and the following independent variables of the study: “gender identity;” “age;” “highest level of education completed;” “household income;” “suicidal ideation;” “satisfaction with oral health status;” and “difficulty accessing dental treatment.”

**Table 3:** Associations between the prevalence measure of the impact of OHRQoL (OHIP-14) on LGBTIQ+ people and the independent variables of the study (n = 464).

Category	n ( )*	Prevalence of impact		Unadjusted OR (95% CI)	p-value

		No impact	Impact		

		n ( )**			
Global	464 (100.0)	310 (66.8)	154 (33.2%)	-	-
Level 1 – General Stressors	-	-	-	-	-
Highest completed level of education					
Up to elementary	23 (5.0)	11 (47.8)	12 (52.2%)	4.12 (1.64-10.36)	0.0026
High school and/or technical	198 (42.7)	123 (62.2)	75 (37.9%)	2.30 (1.38-3.84)	0.0014
Higher/college	90 (19.4)	63 (70.0)	27 (30.0%)	1.62 (0.87-3.01)	0.1271
Graduate	129 (27.8)	102 (79.1)	27 (20.9%)	Ref	
Household income US$					
Less than US$ 783	224 (48.3)	120 (53.6)	104 (46.4%)	4.04 (1.88-8.70)	0.0004
US$ 784 – US$ 1,305	81 (17.5)	58 (71.6)	23 (28.4%)	1.85 (0.78-4.40)	0.1641
US$ 1306 – US$ 1,827	55 (11.8)	45 (81.8)	10 (18.2%)	1.04 (0.38-2.80)	0.9428
US$ 1828 – US$ 2,610	50 (10.8)	44 (88.0)	6 (12.0%)	0.64 (0.21-1.94)	0.4278
More than US$ 2,610	51 (11.0)	42 (82.4)	9 (17.6%)	Ref	
Did not answer / know	3 (0.6)	1 (33.3)	2 (66.7%)	-	
Difficulty accessing dental treatment					
Yes	197 (42.5)	93 (47.2)	104 (52.8%)	5.01 (3.28-7.66)	< 0.0001
No	252 (54.3)	206 (81.8)	46 (18.2%)	Ref	
I never looked for and/or been to the dentist	15 (3.2)	11 (73.3)	4 (26.7%)	1.63 (0.50-5.34)	0.4212
Level 2 – Distal stress processes					
Gender identity					
Cisgender	328 (70.7)	235 (71.7)	93 (28.4%)	Ref	
Transgender	136 (29.3)	75 (55.2)	61 (44.8%)	2.06 (1.36-3.11)	0.0007
Sexual orientation					
Heterosexual	42 (9.0)	25 (59.5)	17 (40.5%)	Ref	
Homosexual	257 (55.4)	182 (70.8)	75 (29.2%)	0.61 (0.31-1.19)	0.1442
Bisexual	96 (20.7)	68 (70.8)	28 (29.2%)	0.61 (0.28-1.29)	0.1941
Asexual	16 (3.4)	10 (62.5)	6 (37.5%)	0.88 (0.27-2.89)	0.8360
Pansexual	33 (7.1)	17 (51.5)	16 (48.5%)	1.38 (0.55-3.47)	0.4885
Other	14 (3.0)	4 (28.6)	10 (71.4%)	3.68 (0.99-13.37)	0.0520
Did not answer / know	6 (1.3)	4 (66.7)	2 (33.3%)	-	
Age (years)					
Up to 27***	240 (51.7)	147 (61.2)	93 (38.8%)	1.69 (1.14-2.50)	0.085**
Over 27	224 (48.3)	163 (72.8)	61 (27.2%)	Ref	
Color/race/ethnicity					
White	247 (53.2)	175 (70.8)	72 (29.2%)	Ref	
Brown	131 (28.2)	84 (64.1)	47 (35.9%)	1.36 (0.87-2.13)	0.1808
Black	68 (14.7)	42 (61.8)	26 (38.2%)	1.50 (0.86-2.64)	0.1534
Yellow/Oriental/Japanese	6 (1.3)	3 (50.0)	3 (50.0%)	2.43 (0.48-12.33)	0.2837
Indigenous	5 (1.1)	3 (60.0)	2 (40.0%)	1.62 (0.26-9.90)	0.6012
Other	7 (1.5)	3 (42.9)	4 (57.1%)	3.24 (0.71-14.83)	0.1302
Suicidal ideation					
No	179 (38.6)	133 (74.3)	46 (25.7%)	Ref	
Yes. I just thought about suicide	146 (31.5)	103 (70.6)	43 (29.4%)	1.21 (0.74-1.97)	0.4509
Yes. I thought about and planned suicide	50 (10.8)	26 (52.0)	24 (48.0%)	2.67 (1.40-5.10)	0.0030
Yes I thought about, planned, and tried suicide	72 (15.5)	38 (52.8)	34 (47.2%)	2.59 (1.46-4.58)	0.0011
Did not answer / know	17 (3.7)	10 (58.8)	7 (41.2%)	-	
Level 3 – proximal stress processes					
Satisfaction with oral health status					
Very satisfied	52 (11.2)	46 (88.5)	6 (11.5%)	Ref	
Satisfied	162 (34.9)	142 (87.6)	20 (12.4%)	1.08 (0.41-2.85)	0.8768
Neither satisfied nor dissatisfied	136 (29.3)	91 (66.9)	45 (33.1%)	3.79 (1.51-9.54)	0.0046
Dissatisfied	82 (17.7)	28 (34.2)	54 (65.8%)	14.79 (5.63-38.83)	< 0.0001
Very dissatisfied	32 (6.9)	3 (1.0)	29 (90.6%)	74.11 (17.18-319.68)	< 0.0001
Believe the dentist is prepared to care for LGBTIQ+ people					
Yes	106 (22.8)	74 (69.8)	32 (30.2%)	Ref	
No	189 (40.7)	123 (65.1)	66 (34.9%)	1.24 (0.74-2.07)	0.4081
Did not answer / know	169 (36.4)	113 (66.9)	56 (33.1%)	0.15 (0.68-1.94)	0.6102

OR: Odds ratio; CI: Confidence interval; Ref: Reference category of the independent variable; ^*^Percentages in the columns; ^**^Percentages in the rows; ^***^Sample median.

In the case of gender identity, the values pointed to a greater impact on the OHRQoL of transgender people when compared to cisgender people, considering that 44.8% of transgender individuals answered ‘frequently’ or ‘always’ to one or more items of the OHIP-14 (prevalence), while for cisgender individuals, this frequency was nearly half (28.3%) (Table 3).

The level of education showed a relationship between lower educational levels and greater prevalence of the impact on OHRQoL. Thus, among those who answered ‘frequently’ or ‘always’ to one or more items of the OHIP-14 (prevalence), 57.1% had no schooling or 52.2% had completed elementary school (Table 3).

With regard to family income, the most vulnerable individuals in terms of OHRQoL were those in the poorest family settings. The frequency of impact among those with incomes below three BMW or US$ 783 (46.4%) was much higher than those who reported income above 10 BMW or US$ 2,610 (17.6%) (Table 3).

As for suicidal ideation, vulnerability was more intense among those who thought about and planned (48.0%) suicide and those who thought about, planned, and tried (47.2%) suicide.

With regard to oral health, the greatest impact on OHRQoL was verified among those who were very dissatisfied with their oral health status (90.6%) and who reported difficulty accessing dental treatment (52.8%) (Table 3).

Next, the hierarchical multiple logistic regression was used to evaluate the variables associated with the outcome OHIP-14-prevalence in the sample. According to the adjusted final model, LGBTIQ+ people who were more likely to feel the impact on their OHRQoL were those who were neither satisfied nor dissatisfied (OR=3.21; 95%CI: 1.26–8.20), dissatisfied (OR = 10.45; 95%CI: 3.86–28.26) or very dissatisfied (OR = 53.93; 95%CI: 12.12–239.93) with their oral health status, and also those who had or have difficulty accessing dental treatment (OR = 2.06; 95%CI: 1.24–3.41) (p < 0.05) (Table 4).

**Table 4:** Results of multiple regression analyses for the predictor variables associated with the OHIP-14-prevalence of LGBTIQ+ people (n = 464).

Category	Model 1		Model 2		Model 3 (final)	

	OR (95%CI)	p-value	OR (95%CI)	p-value	OR (95%CI)	p-value
Level 1 – General stressors						
Household income US$						
Less than US$ 783	2.37 (1.06–5.32)	0.0359	–	–	–	–
US$ 784 – US$ 1,305	1.50 (0.61–3.72)	0.3772	–	–	–	–
US$ 1306 – US$ 1,827	0.84 (0.30–2.36)	0.74428	–	–	–	–
US$ 1828 – US$ 2,610	0.70 (0.22–2.20)	0.5430	–	–	–	–
More than US$ 2,610	Ref	–	–	–	–	–
Did not answer / know	–	–	–	–	–	–
Difficulty accessing dental treatment						
Yes	3.69 (2.32–5.85)	< 0.0001	5.56 (3.54–8.74)	< 0.0001	2.06 (1.24–3.41)	0.0053
No	Ref		Ref		Ref	
I never looked for and/or been to the dentist					0.69 (0.17–2.86)	0.6097
Level 2 – Distal stress processes						
Suicidal ideation						
No	–	–	Ref		–	–
Yes. I just thought about suicide	–	–	1.00 (0.59–1.70)	0.9986	–	–
Yes. I thought about and planned suicide	–	–	2.91 (1.43–5.91)	0.0031	–	–
Yes I thought about, planned, and tried suicide	–	–	2.34 (1.26–4.36)	0.0070	–	–
Did not answer / know	–	–	–		–	–
No	–	–	–	–	–	–
Level 3 – proximal stress processes						
Satisfaction with oral health status						
Very satisfied	–	–	–	–	Ref	
Satisfied	–	–	–	–	1.12 (0.42–2.99)	0.8166
Neither satisfied nor dissatisfied	–	–	–	–	3.21 (1.26–8.20)	0.0146
Dissatisfied	–	–	–	–	10.45 (3.86–28.26)	< 0.0001
Very dissatisfied	–	–	–	–	53.93 (12.12–239.93)	< 0.0001
AIC						
Empty model=560.04	502.56		500.56		461.157	

OR: Odds ratio; CI: Confidence interval.

Considering the findings in Table 3 regarding the greater prevalence of the impact on the OHRQoL of transgender people, some additional exploratory analyses were carried out. Table 5 shows the results of associations between cisgender and transgender people who answered ‘frequently’ or ‘always’ to one or more items of the OHIP–14 for the variables highest level of education completed, household income, suicidal ideation, satisfaction with oral health status, and difficulty accessing dental treatment. There was a significant association between gender identity and suicidal ideation, and satisfaction with oral health status and difficulty accessing dental treatment (p < 0.05) for people whose OHRQoL was affected (OHIP–14 prevalence measure).

**Table 5:** Analysis of the associations between cisgender and transgender persons for the sample with impact on OHRQoL (OHIP–Prevalence) and highest completed level of education, household income, suicidal ideation, satisfaction with oral health status, and difficulty accessing dental treatment (n = 154).

Category	Gender identity		p-value

	Cisgender	Transgender	

	Frequency (%)		
Global	93 (60.4)	61 (39.6)	-
Highest completed level of education			
No schooling	1 (1.1)	3 (4.9)	0.1052*
Elementary	5 (5.4)	7 (11.5)	
High school and/or technical	46 (49.5)	29 (47.5)	
College	18 (19.4)	9 (14.8)	
Graduate	21 (22.6)	6 (9.8)	
^3^Did not answer / know	2 (2.2)	7 (11.5)	
%Household income			
Less than US$ 783	57 (61.3)	47 (77.0)	0.3374*
US$ 784 – US$ 1,305	16 (17.2)	7 (11.5)	
US$ 1306 – US$ 1,827	7 (7.5)	3 (4.9)	
US$ 1828 – US$ 2,610	5 (5.4)	1 (1.6)	
More than US$ 2,610	7 (7.5)	2 (3.3)	
Did not answer / know***	1 (1.1)	1 (1.6)	
Suicidal ideation			
No	36 (38.7)	10 (16.4)	0.0012**
Yes, I just thought about suicide	27 (29.0)	16 (26.2)	
Yes, I thought about and planned suicide	8 (8.6)	16 (26.2)	
Yes, I thought about, planned, and tried suicide	16 (17.2)	18 (29.5)	
Did not answer / know***	6 (6.4)	1 (1.6)	
Satisfaction with oral health status			
Very satisfied	5 (5.4)	1 (1.6)	0.0010**
Satisfied	15 (16.1)	5 (8.2)	
Neither satisfied nor dissatisfied	34 (36.6)	11 (18.0)	
Dissatisfied	30 (32.3)	24 (39.3)	
Very dissatisfied	9 (9.7)	20 (32.8)	
Difficulty accessing dental treatment			
Yes	55 (59.1)	49 (80.3)	0.0009*
No	37 (39.8)	9 (14.8)	
I never looked for and/or been to the dentist	1 (1.1)	3 (4.9)	

^*^Fisher’s exact **test; Chi–square test. ^***^Cases of non–response or ‘don’t know’ were not considered for the application of the hypothesis test.

## Discussion

This study investigated the associations between the OHRQoL of a sample of LGBTIQ+ people and their sociodemographic data, suicidal ideation, self–perception of oral health, and history of dental treatment. To our knowledge, this is the first study to date in Brazil evaluating these aspects in a sample of LGBTIQ+ people, using a hierarchical model of analysis, thereby bringing new evidence for the oral health care of this population.

The prevalence of OHIP–14 impacts in the sample was 33.2%, similar to that of a study conducted with this population in India.^9^ Likewise, psychological discomfort was the OHIP–14 domain with higher prevalence of reported impacts on LGBTIQ+ people in India and Malaysia.^9,10^

Compared to other populations, the prevalence of OHIP–14 impacts (OHIP–prevalence) in the present sample was higher than that verified in the general population in UK (16.%),^31^ Canada (19.5%),^32^ Australian men aged 70 years or older (10%),^33^ and adults in São Leopoldo, Brazil.^34^ However, these percentages were lower than in populations with mental illness, generally greater than 50%,^25^ among Australian people who inject drugs (48%),^35^ and in rural riverine populations in Amazonas, Brazil (44.3 and 70.3).^36^

It was verified in the adjusted final model of regression that the OHRQoL of the individuals was statistically associated with satisfaction with the self–perceived oral health and difficulty accessing dental treatment.

Negative self–perception of oral health status, according to a systematic literature review, was associated with unfavorable social, economic, demographic, psychosocial, and behavioral factors, as well as with poor oral clinical status, and with OHIP–14 scores.^37^ Self–perception of oral health represents an important marker of OHRQoL for populations in many countries in that they affect people throughout their lives, whether due to pain and/or aesthetic issues and/or functional deviations of the stomatognathic system.^37^ According to a study developed among lesbian, gay, and bisexual U.S. adults, subjective measures of oral health were worse in this population compared to those of heterosexual adults.^5^

Difficult access to dental treatment could be associated with worse OHRQoL, as confirmed in previous studies.^9^As for accessibility to dental treatment, there is evidence that the LGBTIQ+ population has less access to health services, including dental services,^1^both in terms of quantity and quality, a fact that could be attributed to the professionals’ lack of preparation and sensitivity to take care of this population, which reiterates inequities in their access to health services.^11,38^In addition, there are some obstacles encountered by these individuals in their access to health services, which is initially characterized by the difficulty and even inaccessibility to the health care network, discriminatory care by the services, embarrassment, prejudiced connotations, or even verbal and/or physical offenses uttered by professionals.^1^

Faced with this harsh reality, it is essential to understand the complexity of the difficulty in accessing health services, including dental care, by the LGBTIQ+ population, as they face issues ranging from their safety until they arrive at a health unit to the training of the entire heath team for the recognition and reception of these people.^1^

LGBTIQ+ is not a homogeneous population; therefore, health disparities exist between sexual orientation groups.^5^Firstly, the frequency of thought and/or planned and/or tried suicide was 54.9% in cisgender persons and 82% in transgender individuals (Table 5). According to a recent systematic review and meta–analysis, transgender people are at a higher risk of experiencing suicidal thoughts during their lifetime compared to other gender minority populations, and almost half of the transgender individuals who have suicidal thoughts commit suicide.^16^ This information is very important for health teams and services.

In the present study, in the total sample, less than half of the participants reported being “very satisfied” or “satisfied” with their oral health status. However, when gender identity was taken into account (Table 5), there was a significant association between the transgender group and satisfaction with oral health status, compared to cisgender individuals. This finding was corroborated by the studies of Prates et al. (2021)^11^ and Soares (2022).^6^ There are several factors that can contribute to these differences, including the greater need felt by transgender people to meet the social expectations about the female figure and what they accept as beautiful, and worse oral health status of transgender individuals.^6,11^

The same differences were observed in the difficulty in accessing dental services. Studies have shown this is a prevalent problem that has become even more critical when transgender individuals are involved, given that transsexuality is still stigmatized and discriminated against by many health professionals and managers of health care services, as well as by the society, and these factors keep these people from seeking treatment in health care settings.^4^Thus, our results are in line with those of previous studies,^5,6^demonstrating that transgender persons ae among the most vulnerable groups in the LGBTIQ+ population.^39^ In order to overcome this problem, it is essential to include content and activities related to the LGBTIQ+ population in the training of future professionals in order to enable them to understand and address the specific needs and demands of these individuals for oral health care.^1,38,40^

This study has some limitations. The data were collected by self–administered questionnaires on an online environment, and that may have limited their completion by those with limited internet access or low computer literacy, thus compromising the sample size and representativeness of the population. Despite adequate effect size, the number of participants was affected by the range of some confidence intervals; therefore, we suggest the use of larger samples in future studies. No clinical exams were performed and, consequently, we were unable to know whether the self–perception of oral health in this population reflects clinical status from their mouths. Therefore, generalizability of the results of this study should be made with caution and future studies should seek to evaluate these aspects in other samples.

The strengths of our study were the sufficient sample size, which was much larger and representative than that of other studies carried out in Brazil (including 5 to 329 respondents).^6,11,12^In addition, we used a validated instrument to investigate OHRQoL (OHIP–14), which allows us to compare our results with those of other studies that have adopted the same criterion. Moreover, the difficulty in investigating LGBTIQ+ individuals is noteworthy, considering that most of them do not reveal their sexual orientation easily because of social restrictions, and also that they have lower access to and use of dental services, especially transgender persons.

The findings of this study highlight a key element that could shape more effective public policies for oral health, thus having a greater impact on the well–being of the LGBTIQ+ population, which is underrepresented in studies, training, and dental care.

## Conclusion

OHRQoL of the investigated Brazilian LGBTIQ+ population showed associations with self–perception of oral health and difficulties in accessing dental treatment.
